# The relationships among learning engagement, continuous improvement attitude and creativity performance: a study based on a C-STEAM course

**DOI:** 10.3389/fpsyg.2026.1738042

**Published:** 2026-02-18

**Authors:** Chen Qian, Jian-Hong Ye, Lin Zhang, Lei Wang

**Affiliations:** 1 College of Design and Innovation, Zhejiang Normal University, Jinhua, China; 2 Faculty of Education, Beijing Normal University, Beijing, China; 3 National Institute of Vocational Education, Beijing Normal University, Beijing, China; 4 School of Education, Binzhou Polytechnic, Binzhou, China; 5 Business School, Jinhua University of Vocational Technology, Jinhua, China

**Keywords:** art design, Chinese culture, creativity performance, C-STEAM education, design education, interdisciplinary education, Shangshan Culture, traditional Chinese culture

## Abstract

**Introduction:**

In the context of new quality productive forces in China, promoting innovative transformation of local culture by using high levels of technology has become a hot topic. However, there have been few empirical studies exploring the influence of interdisciplinary cultural inheritance and innovation on students’ learning outcomes. Hence, in this study, we recruited 130 students from a higher vocational college in China, for whom a local culture-based science, technology, engineering, art and mathematics (C-STEAM) art and design course was established.

**Methods:**

By adopting a single-group pre–post intervention design, a model comprising seven hypotheses based on learning engagement theory was developed. To understand the cognitive and emotional responses of students after taking the course, the learning engagement scale and continuous improvement attitude scale were utilized. Creativity performance analysis was used as a measuring tool to gain insights into the changes in students’ knowledge and skills, as well as the overall learning effectiveness after their participation in the course.

**Results:**

According to the research results, receiving 72 h of theoretical and practical packaging training positively influenced the students’ interdisciplinary literacy and continuous improvement attitudes. Students with higher learning engagement showed better continuous improvement attitude and creativity performance, and students with better continuous improvement attitude had better creativity presentation.

**Discussion:**

By maximizing the close integration of interdisciplinary knowledge into the teaching subject, this study promotes the high-precision cultivation of art talents, provides support for the academic community from an art design perspective, and innovates the inheritance and development of Chinese culture.

## 1 Introduction

Culture is both the root and means of design, and can subtly influence and even decide the growth of students. In higher vocational education, only by enhancing the cultural literacy of vocational and technical talents can the creativity of students be improved and national modernization and high-quality development be realized, thus promoting comprehensive national strength and competitiveness. China has more clearly set the grand goal of creating a new culture in a new era (Lin et al., 2024) and of building a modern Chinese civilization (The Central People’s Government of the People’s Republic of China, 2023). Currently, new quality productive forces play a leading role in Chinese cultural innovation (Wan et al., 2024).

As a novel form of productivity dominated by high-technology innovation (Zhou and Li, 2024), new quality productive forces aim to activate the vitality of Chinese culture and enable cultural inheritance through the cross-integration of frontier scientific and technological innovations. Accordingly, in the context of new quality productive forces, the demand for high-quality individualized design is growing rapidly, requiring innovative breakthroughs in art design. In addition to technological innovation, the innovative transformation of culture is also very important, as it endows creation with a higher quality productive value.

Since the promotion of cultural education in colleges and universities, the integration of excellent traditional Chinese culture into curriculum teaching has mostly been concentrated in courses such as ideology, politics, mathematics and foreign languages, and the majority of teaching designs have been based on existing classroom examples (Sun et al., 2023), which lack independent innovation modes adapted to local conditions (Cui et al., 2025), and thus hinder sustainable development (Qian et al., 2022). Consequently, students’ knowledge is isolated, scattered, abstracted, less connected to local culture (Zhan et al., 2023), disconnected from daily life and less involved (Jiang and Chen, 2026), which is detrimental for students to maintain their attention and strengthen their sense of excellent local cultural and historical identity.

Hence, on the basis of the interdisciplinary STEAM (Science, Technology, Engineering, Art and Mathematics) education model, Zhan et al. (2022) proposed the C-STEAM model, which is oriented toward local cultural inheritance and focuses on cultivating students’ comprehensive literacy and innovative inquiry ability. In recent years, the STEAM education model combining courses and design project instruction has become the mainstream (Fan and Ye, 2020), especially in design courses such as fashion product design, textile design and packaging design (Qian et al., 2023). Against this background, the present study focused on a culture-based STEAM (C-STEAM) packaging design course implemented in a higher vocational context to explore the psychological mechanisms underlying students’ creativity development.

Excellent traditional culture reflects a nation’s spiritual will and value orientation (Ruan et al., 2024; Zhan et al., 2022), embodying the ethics and values universally recognized by local people (Li and Wang, 2023; Zhao et al., 2021), as well as their profound ideological connotations, artistic value and esthetic habits (Wang et al., 2024; Ye et al., 2023). In China, Shangshan Culture features the most influential cultural relics with Chinese characteristics and local significance (Qian and Wang, 2022). One of the important windows for understanding China around 10,000 years ago and the origin and formation of Chinese culture for people around the world is the Shangshan Site, which is known as the cradle of the world’s painted pottery civilization, the birthplace of Chinese farming villages, and the origin of the world’s rice cultivating civilization (Jiang, 2013). The features of pottery, stone tools, wooden pile dwellings, rice remains and rice seedling nurseries unearthed from Shangshan, as well as the wisdom and cultural thoughts contained in the scientific material selection, ware polishing, dwelling construction, rice cultivation, ware decoration and tool measurement of Shangshan ancestors are all consistent with the multi-sensory experience of C-STEAM education. Thus, Shangshan Culture was integrated into a STEAM course, and through the research, refining and observation of local culture and its integration into creative packaging design, students were able to find and solve problems in actual projects, thereby allowing improvements in their hands-on ability and creative presentation skills.

Learning engagement refers to a kind of mental state shown by learners during their learning process (Fredricks et al., 2004), which is the quality of emotional experience and the expression of positive behavior. Thus, learner engagement is considered an important influencing factor in course learning and one of the major conditions for achieving meaningful learning. It holds that the basis for learners to engage in learning tasks is to satisfy their demand for competence. Such competence can be regarded as a motivation source, which prompts students to devote more effort and emotion to their studies through actual projects or tasks assigned by schools. In this study, learning engagement was defined as the degree of emotional, behavioral and cognitive investment made by learners when they participated in the C-STEAM-integrated Packaging Design course, and the process of positive attitude and active response they displayed during their participation in the course.

Most students expect to assume responsibility for their positive behavior during course learning, believing it to be a very affirmative thing. Continuous improvement is regarded as a key auxiliary condition for continuous progress in course learning since it is a philosophy which can increase success and reduce failure (Ye et al., 2020). An attitude can be considered an evaluative reaction to a person, an object or an abstract idea (Albarracin and Shavitt, 2018) that reflects the holistic concept. This means that attitudes summarize different types of consultations about people, subjects and issues (Haddock and Maio, 2019). A good piece of work often needs repeated communication and continuous improvement during its design in order to facilitate its gradual perfection; design is therefore closely correlated with continuous improvement. The concept of continuous improvement attitude in this study refers to the learner’s willingness to continuously improve their work throughout the packaging design and production processes.

In creative design courses, creativity performance is considered one of the measures of learning effectiveness or course evaluation. As a behavioral expression of creative potential (Zhu et al., 2022), creativity performance enables individuals to come up with more useful or novel ideas from their own perspective (Wang and Lau, 2022), develop creative work, and invest their ability and energy into novel useful ideas (Amro et al., 2023). The generation of individual creativity performance is the result of the interaction between the human body and its environment (Choi, 2020). The outcomes of creativity performance can be new products, processes and technologies, or new ideas, concepts and theories (Zhao et al., 2021). In this study, creativity performance is defined as the thinking process of learners during a packaging design task, and the subsequent production of an original and practical design work by flexibly applying learned knowledge.

In summary, the integration of C-STEAM education with a packaging design course can be of great help. However, existing studies concerning C-STEAM education have tended to focus on K-12 (Jiang and Chen, 2026). There are few studies concerning multicultural integration into higher education, especially higher vocational education, and the factors influencing the emotions and attitudes of students taking C-STEAM art design courses have never been discussed. This study adopted a single-group pre–post intervention design—a practical approach when participant numbers are limited (Fan and Ye, 2022). It examined the intersection of cultural education, STEAM, and art design within a higher vocational context, developing a Shangshan Culture-based packaging design course. Using learning engagement theory, the study analyzed relationships among engagement, attitudes, creativity, and knowledge acquisition, proposing a model to understand students’ cognitive and emotional responses. Learning outcomes were compared to assess changes in knowledge, skills, and overall course effectiveness.

## 2 Theoretical basis and research hypotheses

### 2.1 Engagement theory

Engagement theory targets the energy and time spent by students in educational activities, which is the process of thinking, feeling and acting while learning (Kuh, 2001). The physical energy and time invested in learning activities by students can promote their expectation of success. Student engagement was theorized by Gonida et al. (2009) as positive behaviors including devotion and persistence, as well as positive emotions such as curiosity, novelty, enjoyment, fun and low anxiety experienced in classroom assignments. The learning engagement theory directly focuses on students’ behavior and motivation. When students gain successful experience in learning activities through self-investment and efforts, and obtain satisfaction by demonstrating skills for performing learning tasks, they are likely to exhibit continuous behavioral engagement. Currently, learning engagement theory is one of the key frameworks in educational research as it helps explain how students produce corresponding learning outcomes after taking a course. Thus, it can help interpret how students’ involvement in a course affects their cognitive and emotional changes, thereby further promoting the research results.

### 2.2 Research hypotheses

#### 2.2.1 Correlation between learning engagement and continuous improvement attitude

Learning engagement plays a crucial role in course learning. Studies have shown that the more time, energy and emotion students devote to courses with higher effort requirements (Lu et al., 2023), the stronger their perseverance when faced with problems (Wang and Eccles, 2011). According to recent findings, students’ involvement in design courses is regarded as one of the factors that can continuously improve the quality management of design works (Ye et al., 2020). Emotional engagement is not only an expression of learning attitude, but also the emotional attitude toward and affective recognition of courses, teachers and design works (Danna et al., 2023). Behavioral engagement is reflected in the effort level and performance status of students in classroom learning, which shows a state of persistent behavior (Kuh, 2009). Cognitive engagement is a process of regulating course learning by asking questions, completing tasks and regulating oneself via metacognitive strategies (Ali et al., 2022). Hence, this study explored the relationship between students’ learning engagement and their continuous improvement attitude in the packaging design scenario, and proposed the following hypotheses:

*H1*: Cognitive engagement has a positive effect on continuous improvement attitude.

*H2*: Emotional engagement has a positive effect on continuous improvement attitude.

*H3*: Behavioral engagement has a positive effect on continuous improvement attitude.

#### 2.2.2 Correlation between learning engagement and creativity performance

Learning engagement is theorized as positive behaviors such as devotion and persistence shown by students in course learning, as well as positive emotions such as enjoyment, curiosity and low anxiety experienced during coursework (Ye et al., 2020). The basis for students’ engagement in learning tasks is to meet the demand for competence, which can be regarded as a source of motivation (Ali et al., 2022). The quantity and quality of students’ engagement in creative activities are closely and positively correlated with their learning outcomes (You, 2022), while students’ creativity performance and personal development level can be directly related to their engagement (Deng et al., 2022; Hong et al., 2020a,b). That is, the more time and energy students spend on creative activities requiring greater efforts, the more benefits they will receive (Lu et al., 2023). Thus, this study explored the relationship between students’ learning engagement and their creativity performance in the packaging design scenario, and proposed the following hypotheses:

*H4*: Cognitive engagement has a positive effect on creativity performance.

*H5*: Emotional engagement has a positive effect on creativity performance.

*H6*: Behavioral engagement has a positive effect on creativity performance.

#### 2.2.3 Correlation between continuous improvement attitude and creativity performance

Continuous improvement is a refinement principle that can increase success and reduce failure (Holtskog, 2013). Relevant studies have confirmed that refinements generated by continuous improvement can directly affect the quality, products and manufacturing process (Su and Linderman, 2016), while other specific capabilities (innovation, creativity) also positively affect quality (Peng and Chen, 2023). Attitude, on the other hand, refers to an evaluative judgment of the holistic concept, which summarizes different types of consultations about issues, people and subjects (Haddock and Maio, 2019). Thus, it can be speculated from the relevant research of Su and Linderman (2016) that continuous improvement attitude is positively correlated with creativity performance. In this study, students’ continuous improvement attitude during packaging design is discussed, and its relationship with creativity performance was analyzed, based on which the following hypothesis was proposed:

*H7*: Continuous improvement attitude has a positive effect on creativity performance.

### 2.3 Research model

**Figure 1:**
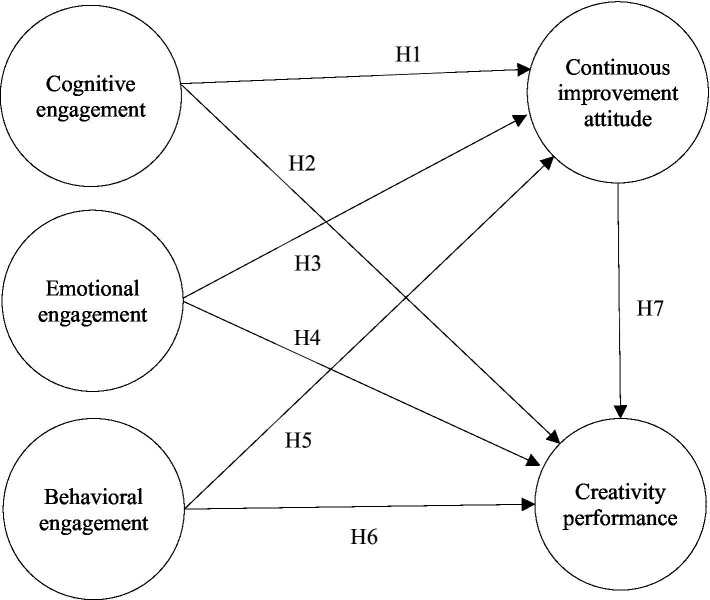
Research model.

In addition to in-depth understanding of course learning effectiveness, it is also important to identify the factors influencing students’ course learning intentions, behaviors and outcomes, such as motivation, persistence and academic achievement. Hence, based on the learning engagement theory, in this study, we collated relevant literature concerning learning engagement, continuous improvement attitude and creativity performance to identify five research variables (cognitive engagement, emotional engagement, behavioral engagement, continuous improvement attitude and creativity performance), based on which a research model of creativity performance for art design major students in Chinese higher vocational colleges was developed, as shown in Figure 1.

## 3 Methods

### 3.1 Course design

#### 3.1.1 Course connotation

This study implemented a C-STEAM packaging design course by integrating Shangshan Culture into a higher vocational art and design curriculum. Packaging design was selected as the instructional context because it requires the coordinated application of interdisciplinary knowledge, including materials, structure, technology, esthetics, and measurement, which closely aligns with the STEAM framework. Through culturally grounded design tasks, students were encouraged to observe, refine, and reinterpret local cultural elements, and apply them to creative packaging projects. This innovative Packaging Design course integrates STEAM education and Shangshan Culture. Taking actual commodities as the topic, it deeply fuses excellent local culture with science, technology, engineering, art and mathematics, guiding students to better fulfill the needs of intelligent, quality-oriented and personalized modern Chinese design by learning interdisciplinary theoretical knowledge and practical skills (Hong et al., 2020a,b). The course is adapted to China’s design development in the context of new quality productive forces, which helps students grow into innovative and cooperative high-quality skilled talents with critical thinking who can understand and inherit culture.

On the basis of the textbook, Packaging Design, edited by Liu et al. (2020), this study practiced the cyclic continuous operation of basic curriculum construction, teaching practice and achievement promotion by dividing the course into four units. The course lasts a total of 72 h (9 weeks), and the teaching objectives (see Supplementary material S1) and teaching design (see Supplementary material S2) are formulated in detail. The course progress is acquired every week as planned, the design works are commented on in a timely fashion, and teaching assistance is given in real time. From shallower to deeper, students are able to thoroughly understand the principles of packaging design and production, with an emphasis on cultivating their ability to independently design packaging structures and decorations by applying interdisciplinary knowledge based on local culture.

#### 3.1.2 Intervention implementation

**Figure 2:**
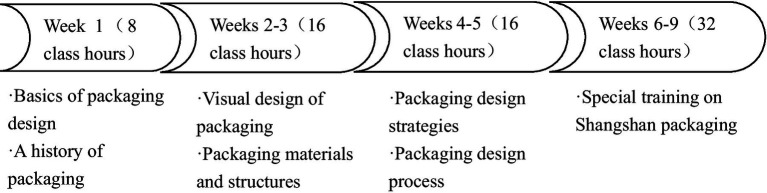
Course planning chart.

The intervention was implemented as a 9-week (72-h) design-based course organized into four sequential units. Instructional activities combined instructor-led lectures and demonstrations, guided studio practice, and platform-supported self-learning. In the initial stage, students were introduced to fundamental principles of packaging design and to a local cultural context through instructor-prepared learning materials, with explicit mapping of cultural interpretation, material knowledge, production processes, structural design, visual expression, and size specification to the C-STEAM components. During the middle stage, students completed stepwise tasks on visual communication and packaging materials and structures, supported by recorded micro-lectures and weekly formative feedback; incremental deliverables (e.g., visual drafts, structural sketches/dielines, and mock-ups) were submitted and refined through instructor critique and targeted one-to-one guidance when needed. In the final stage, students undertook a capstone project involving the design of a serialized packaging set, following a complete workflow from problem definition and user analysis to cultural element extraction, structural prototyping, digital refinement, iterative optimization, and in-class presentation of the final design rationale (Figure 2).

#### 3.1.3 A design example

Table 1 presents the packaging design of a mung bean cake product from Jinhua, Zhejiang, China, where input and creation are accomplished using the digital panel in Auto CAD, Photoshop and Baoxiaohe. The packaging comprises a paper box with a horizontal clamshell structure. In the main visual diagram of packaging decoration, the mung bean, an agricultural product of the Shangshan Site, is personified with reference to the image of “Shangshan Xiaobai” (Qian et al., 2022). This personified cartoon character leads the consumers into the Shangshan Site, and the pottery, rice, vegetables and fruits characteristic of the Shangshan Culture are skillfully combined with the geographical environment.

**Table 1:** Jinhua green bean cake packaging design.

Expanded view	Finished product	Design draft
Design draft	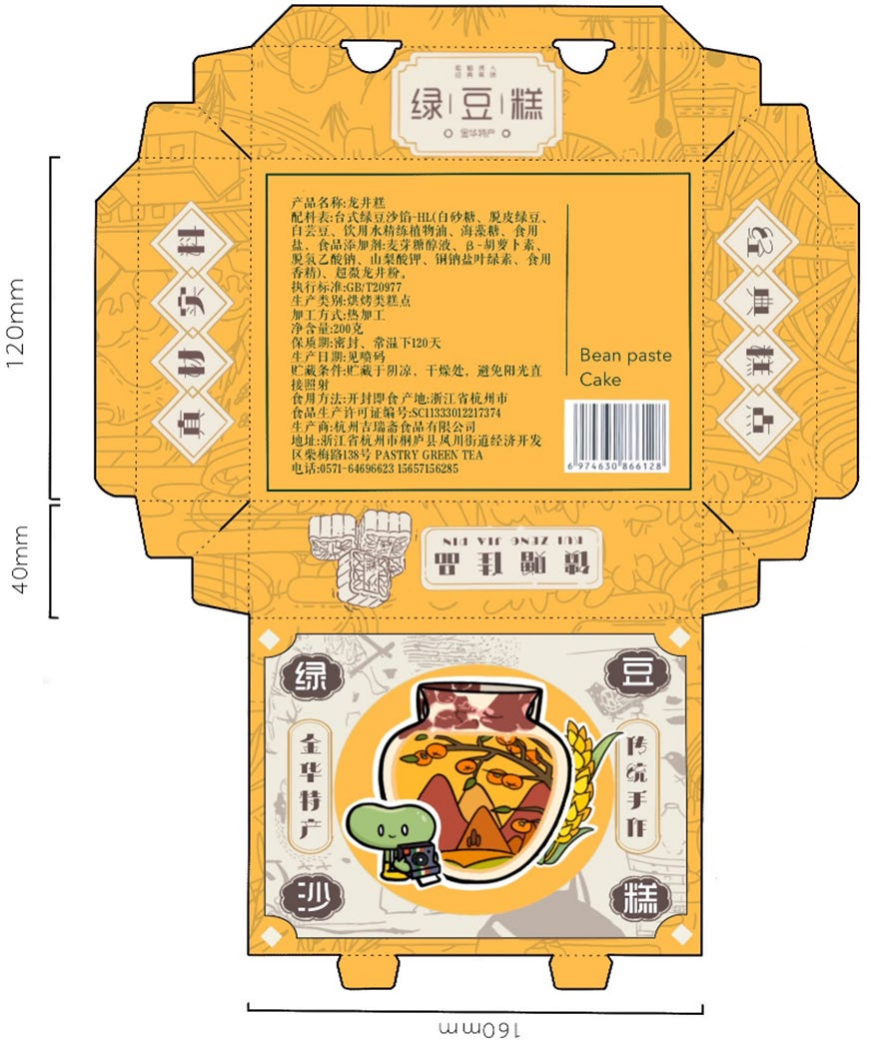	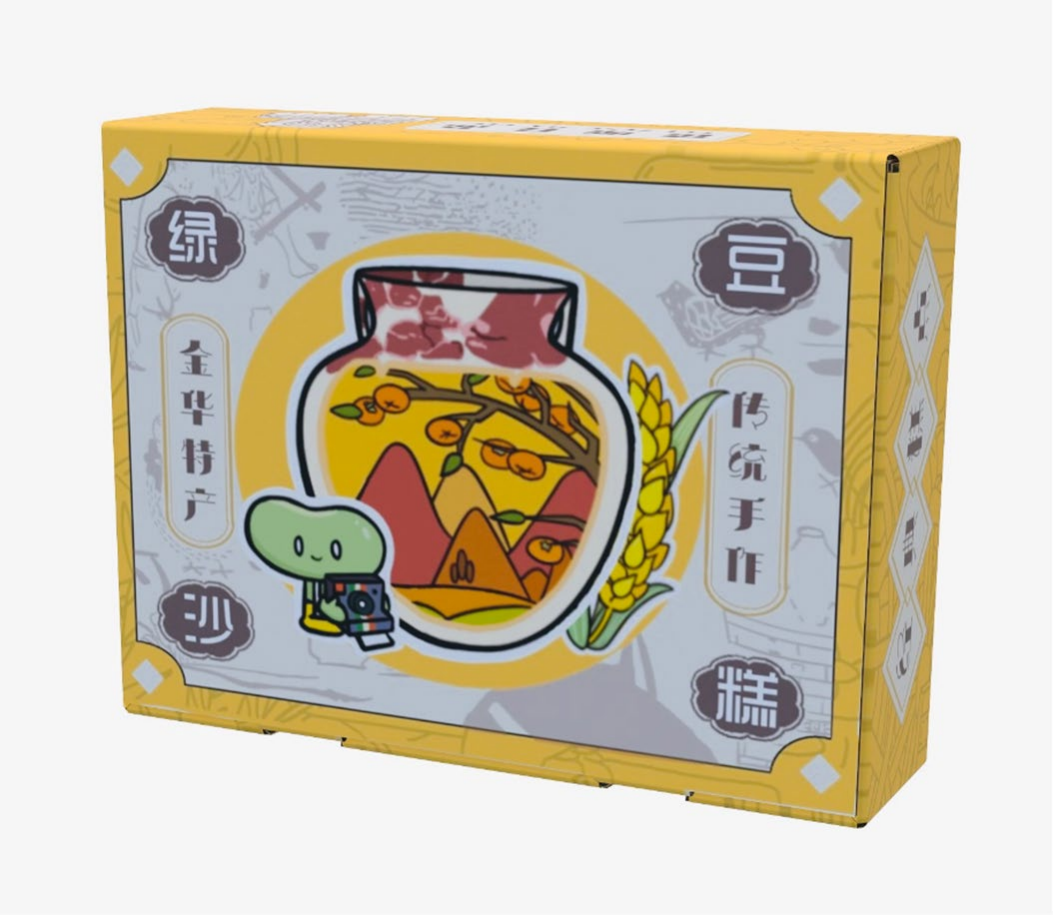

### 3.2 Process and participants

The purpose of this study was to explore the establishment of the Shangshan culture-based STEAM art design course in Chinese higher vocational colleges, as well as its teaching effectiveness. Convenience sampling was used. Specifically, we recruited an intact cohort consisting of all students enrolled in the targeted Packaging Design course at a higher vocational college in China. The course was delivered to three sophomore classes in the art and design major during the intervention semester. All enrolled students were invited to participate, and participation was voluntary based on written informed consent. Therefore, participants were not randomly selected; rather, the sample represents a course-based cohort accessible to the researchers. The inclusion criteria were enrollment in the Packaging Design course and attendance throughout the course period. After data screening, 130 valid questionnaires were retained for analysis. The participants’ mean age was 21.42 years, and their background characteristics are reported in Table 2.

**Table 2:** Basic information of participants.

Variable	Category	Frequency	Percentage (%)
Gender	Female	86	66.15
	Male	44	33.85
Education	Majored in art design in senior high school	54	41.54
	Never majored in art design in senior high school	76	58.46
Course	Had taken a STEAM course	8	6.15
	Had never taken a STEAM course	122	93.85
Learning	Had learned about Shangshan Culture	10	7.70
	Had never learned about Shangshan Culture	120	92.30

### 3.3 Control variables

To reduce potential confounding effects arising from individual background differences, several demographic and learning-related variables were included as control variables in the structural model. Specifically, gender (0 = female, 1 = male), prior art-design learning background (0 = no, 1 = yes), prior STEAM learning experience (0 = no, 1 = yes), and prior knowledge of Shangshan Culture (0 = no, 1 = yes) were controlled for in the PLS-SEM analysis. These control variables were selected based on prior research indicating that demographic characteristics and prior learning experiences may influence students’ creative performance. Specifically, gender differences in creative performance have been well documented in meta-analytic research (Hora et al., 2022). In addition, prior engagement in art and design learning contexts has been shown to foster creative thinking through open-ended and studio-based pedagogies (Sawyer, 2017). Moreover, participation in STEAM-based curricula has been empirically linked to enhanced scientific creativity among students (Tran et al., 2021). Accordingly, gender, prior art/design learning experience, prior STEAM learning experience, and prior cultural knowledge were included as control variables to reduce potential confounding effects.

### 3.4 Analytical strategy (PLS-SEM)

Partial Least Squares Structural Equation Modeling (PLS-SEM) was employed to test the hypothesized relationships among the latent variables. PLS-SEM is a variance-based analytical approach widely used in education and psychology research, particularly when sample sizes are relatively small and the research model is exploratory in nature. Given the sample size of 130, PLS-SEM was considered appropriate for this study. Data analysis was conducted using SmartPLS 3. Prior to hypothesis testing, reliability and validity of the measurement model were assessed.

### 3.5 Research tools

#### 3.5.1 Learning engagement

To measure learning engagement, the scale developed by Ye et al. (2020) was modified (see Appendix 3). The modified 5-point scale comprised 25 items in three dimensions. The reliability and validity were acceptable, with Cronbach’s α values ranging between 0.83 and 0.89, CR values ranging between 0.83 and 0.89, and AVE values ranging between 0.55 and 0.68. The scale was applied to measure students’ perceptions of learning engagement in the three dimensions during the Packaging Design course.

#### 3.5.2 Continuous improvement attitude

The continuous improvement attitude scale was adapted from that of Ye et al. (2020), and comprised a total of eight items. With a Cronbach’s α of 0.94, a CR of 0.94 and an AVE of 0.80, this scale assessed participants’ perceptions of continuous improvement attitude during the design and production of their works.

#### 3.5.3 Creativity performance

The evaluation of creativity performance adopted the indicators formulated by Li and Guo (2018). After the packaging design experiment, the students’ design works were scored by five experts, including three full-time art design teachers, one corporate part-time teacher and one local culture expert, of whom three had senior titles. The evaluation criteria covered seven dimensions (novelty, suitability, technicality, imagination, esthetics, emotionality and comprehensive impression) (Li and Guo, 2018), each of which was scored from 1 to 5 points. The evaluation score for creativity performance was the average of the seven dimensions.

### 3.6 Measurement model assessment

The measurement model was assessed to examine the reliability and validity of the latent constructs prior to testing the structural model. Internal consistency reliability was evaluated using Cronbach’s α and composite reliability (CR). All constructs demonstrated satisfactory reliability, with Cronbach’s α and CR values exceeding the recommended threshold of 0.70.

Convergent validity was assessed by examining factor loadings and average variance extracted (AVE). All retained items exhibited factor loadings above 0.50, and AVE values for all constructs exceeded the recommended minimum value of 0.50, indicating adequate convergent validity. During this process, items with low factor loadings were removed to improve the measurement quality.

Discriminant validity was evaluated using the heterotrait–monotrait (HTMT) ratio of correlations. All HTMT values were below the conservative threshold of 0.85, suggesting satisfactory discriminant validity among the constructs.

Overall, the measurement model demonstrated adequate reliability and validity, supporting its suitability for subsequent structural model analysis.

## 4 Results

Partial Least Squares Structural Equation Modeling (PLS-SEM) was used to examine the relationships among learning engagement, continuous improvement attitude, and creativity performance. Prior to hypothesis testing, the distributions of the composite scores were screened using skewness and kurtosis statistics to ensure no severe departures from normality; therefore, mean-based descriptive statistics are reported.

### 4.1 Reliability and validity analysis

General validation analysis was tested according to the criterion of internal validity depending on factor loading (>0.50), as proposed by Hair et al. (2014). Accordingly, the cognitive engagement dimension was reduced from eight to seven items, emotional engagement from nine to four, behavioral engagement from nine to seven, and continuous improvement attitude from eight to six items.

For good reliability, Cronbach’s α should be greater than 0.7 (Nunally, 1978) to ensure internal consistency reliability, Composite Reliability (CR) should be greater than 0.6 (Anderson and Gerbing, 1988) and have convergence validity, and Average Variance Extracted (AVE) and Factor Loading (FL) values should be greater than 0.5 (Hair et al., 2014). According to the analytical results of this study, Cronbach’s α values of the various dimensions ranged between 0.87 and 0.95, and CR values ranged between 0.88 and 0.95 (see Table 3), all of which met the recommended standards.

**Table 3:** Reliability and validity analysis.

Construct	Mean	*SD*	α	CR	AVE	FL
	3.79	0.68	>0.70	>0.70	>0.50	>0.50
Cognitive engagement	3.84	0.68	0.93	0.92	0.64	0.73 ~ 0.84
Emotional engagement	3.79	0.68	0.87	0.88	0.60	0.67 ~ 0.84
Behavioral engagement	3.9	0.70	0.93	0.94	0.70	0.79 ~ 0.87
Continuous improvement attitude	3.74	0.44	0.95	0.95	0.76	0.72 ~ 0.93

All variables were measured using 5-point Likert-type scales. SD, standard deviation.

In terms of validity, it was suggested by Hair et al. (2014) that convergence validity can only be ensured when AVE and FL both exceed 0.5. According to the analytical results of this study, the AVE and FL values ranged between 0.64 and 0.73 ~ 0.84, respectively, for the cognitive engagement dimension (Table 3), between 0.60 and 0.67 ~ 0.84, respectively, for the emotional engagement dimension, between 0.70 and 0.79 ~ 0.87, respectively, for the behavioral engagement dimension, and between 0.76 and 0.72 ~ 0.93, respectively, for the continuous improvement attitude dimension. All the values exceeded 0.50 for all dimensions, indicating convergence validity.

The Heterotrait-Monotrait Ratio (HTMT) was used to test the discriminant validity of the latent variables. The results (see Table 4) showed that the HTMT ratios among cognitive engagement, emotional engagement, behavioral engagement, continuous improvement attitude, and creativity performance ranged from 0.62 to 0.80, all of which were below the strict cutoff value of 0.85 (Hair et al., 2014). This indicates that the dimensions of each latent variable had clear boundaries and good conceptual distinctiveness, and the discriminant validity of the measurement model was satisfactory, meaning it could be used for subsequent structural model analysis.

**Table 4:** Heterotrait-Monotrait ratio.

Latent variable	Cognitive engagement	Emotional engagement	Behavioral	Continuous improvement attitude	Creativity performance
Cognitive engagement	1.000	–	–	–	–
Emotional engagement	0.723	1.000	–	–	–
Behavioral engagement	0.751	0.689	1.000	–	–
Continuous improvement attitude	0.786	0.712	0.803	1.000	–
Creativity performance	0.645	0.618	0.667	0.692	1.000

### 4.2 Creativity performance analysis

The average score for creativity performance of the Chinese higher vocational students was 3.74, with the highest score being 4.55 and the lowest being 2.85, as shown in Table 5.

**Table 5:** Creativity performance.

Descriptive statistics of the study variables	*M*	*SD*	Med.	Min	Max
Creativity performance	3.74	0.44	3.80	2.85	4.55

### 4.3 Path analysis

**Figure 3:**
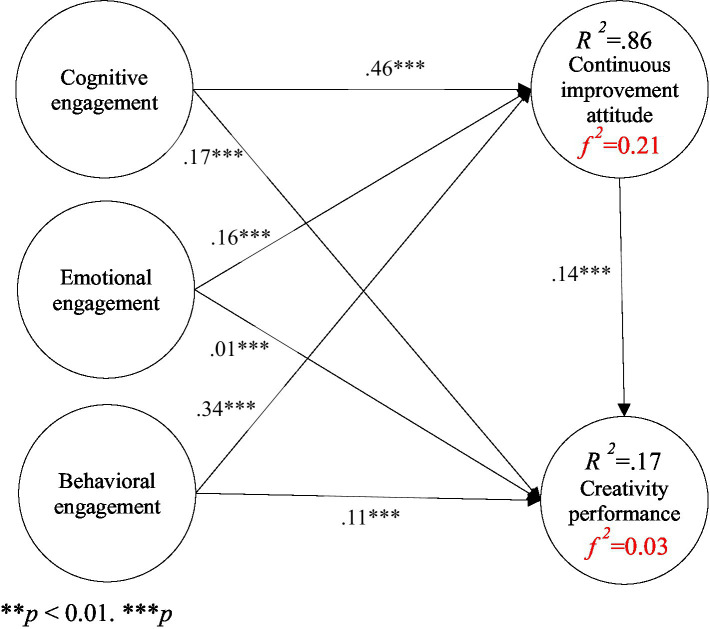
Verification of the research model. **p < 0.01, ***p < 0.001.

The results of the path analysis are summarized in Tables 6, 7 and are illustrated in Figure 3. Based on the learning engagement theory, this study explored the relationships among cognitive engagement, emotional engagement, behavioral engagement, continuous improvement attitude and creativity performance. The analytical results showed that cognitive engagement was positively correlated with continuous improvement attitude (β = 0.46, *p* < 0.001); emotional engagement was correlated with continuous improvement attitude (β = 0.16, *p* < 0.001); behavioral engagement was positively correlated with continuous improvement attitude (β = 0.34, *p* < 0.001); cognitive engagement was positively correlated with creativity performance (β = 0.17, *p* < 0.001); emotional engagement was positively correlated with creativity performance (β = 0.01, *p* < 0.001); behavioral engagement was positively correlated with creativity performance (β = 0.34, *p* < 0.001); and continuous improvement attitude was positively correlated with creativity performance (β = 0.14, *p* < 0.001).

**Table 6:** Indirect effect value.

Path	Independent variable → Mediator β value	Mediator → Dependent variable β value	Indirect effect value
Cognitive Engagement → Continuous improvement Attitude → Creativity Performance	0.817	0.782	0.639
Emotional Engagement → Continuous Improvement Attitude → Creativity Performance	0.835	0.782	0.653
Behavioral Engagement → Continuous Improvement Attitude → Creativity Performance	0.875	0.782	0.684

**Table 7:** Results of regression analyses and hypothesis testing.

Hypothesis	Path	β	*t*-value	*p*-value	Result
H1	Cognitive engagement → Continuous improvement attitude	0.817	22.756	*p* < 0.001	Supported
H2	Emotional engagement → Continuous improvement attitude	0.835	24.369	*p* < 0.001	Supported
H3	Behavioral engagement → Continuous improvement attitude	0.875	29.040	*p* < 0.001	Supported
H4	Cognitive engagement → Creativity performance	0.738	17.567	*p* < 0.001	Supported
H5	Emotional engagement → Creativity performance	0.773	19.563	*p* < 0.001	Supported
H6	Behavioral engagement → Creativity performance	0.796	21.127	*p* < 0.001	Supported
H7	Continuous improvement attitude → Creativity performance	0.782	20.125	*p* < 0.001	Supported

β = standardized regression coefficient. All coefficients were estimated using multiple regression analysis in SPSS.

The three dimensions of learning engagement (cognitive, emotional, and behavioral) explained 86% of the variance in continuous improvement attitude, with a corresponding effect size (f^2^) of 0.21; meanwhile, cognitive, emotional, and behavioral engagements explained 17% of the variance in creativity performance, with an effect size (f^2^) of 0.03.

The indirect effect analysis in Table 6 shows that the indirect effect values of cognitive engagement, emotional engagement, and behavioral engagement on creativity performance via continuous improvement attitude were 0.64, 0.65, and 0.68, respectively. The hypothesis testing results in Table 7 indicate that the standardized regression coefficients (β) of all seven research paths were significant (with *t-*values ranging from 17.57 to 29.04, *p* < 0.001), and all research hypotheses (H1-H7) were supported.

To examine the robustness of the proposed model, background variables were included as control variables in the PLS-SEM analysis. The results showed that none of the control variables had a substantial effect on creativity performance, and the significance and direction of the hypothesized paths remained unchanged. This suggests that the relationships among learning engagement, continuous improvement attitude, and creativity performance were robust after controlling for individual background differences.

## 5 Discussion

In this study, learning engagement, continuous improvement attitude and creativity performance were regarded as cognitive and emotional factors that affect C-STEAM course learning. The more time, energy and emotion students devote to learning, the stronger their learning persistence and, accordingly, the higher their quality requirements for design works. The attitude of continuous design optimization is conducive to promoting students’ creativity performance. Cognitive engagement refers to students’ application of a cognitive strategy in course learning. Emotional engagement is considered students’ inner sense of belonging and identity in the face of a course, while behavioral engagement refers to students’ degree of behavioral involvement in the course learning process. Continuous improvement attitude is defined as students’ willingness to continuously improve their works during the special project production process. Creativity performance is defined as students’ thought processes while working on a special project, as well as their integration of interdisciplinary knowledge for designing personalized works.

### 5.1 Learning engagement is positively correlated with continuous improvement attitude

According to the results of this study, learning engagement had a positive correlation with continuous improvement attitude, so H1, H2, and H3 were verified, showing agreement with the results of Alemayehu and Chen (2023). Learning engagement may require a greater degree of continuous improvement than ordinary creative activities, especially in today’s complex and changing environment, where people are increasingly aware of the need for continuously improving their works and processes (Haddock and Maio, 2019). From this point of view, students who invest more time, energy and emotion have a better attitude toward continuous improvement. The results of this study suggest that learning engagement has a positive effect on continuous improvement attitude.

### 5.2 Learning engagement is positively correlated with creativity performance

According to the results of this study, learning engagement has a positive correlation with creativity performance, so H4, H5 and H6 were verified, showing consistency with the conclusion of Qian et al. (2022). When students’ cognition, emotion and behavior encourage them to invest more energy and time in creative activities, they can achieve success. In this process, they show fully satisfactory skills for performing learning tasks, and are prone to continuously engage after repeated endeavors (Danna et al., 2023). Thus, the more time, energy and emotion that are invested, the greater their influence on creativity performance.

### 5.3 Continuous improvement attitude is positively correlated with creativity performance

The results of this study showed that continuous improvement attitude was positively correlated with creativity performance, so H7 was verified, showing agreement with the finding of Su and Linderman (2016). Students’ higher level of engagement in activities will contribute to the implementation of continuous improvement plans and the development of a continuous improvement culture (Peng and Chen, 2023). To pursue the perfection of design works, and to increase success and reduce failure, students need to persistently participate in practices and constantly propose improvement suggestions to actively solve problems. The generated improvement attitude directly influences the outcome performance. Thus, the better the continuous improvement attitude, the greater its effect on creativity performance.

## 6 Conclusion and suggestions

### 6.1 Conclusion

In the context of new quality productive forces, this study innovatively established a C-STEAM art design course with local characteristics for higher vocational colleges by combining China’s national conditions. Through the integration of Shangshan Culture into the packaging design course, the relationship among learning engagement, continuous improvement attitude and creativity performance was discussed, based on which a novel research model was proposed to understand the cognitive and emotional responses of students after taking the course. The importance and educational significance of teaching C-STEAM in art design were verified. In this study, a 72-h Packaging Design course experiment was conducted involving Chinese vocational art design students. By fully integrating interdisciplinary knowledge, these students could apply their multidisciplinary knowledge of science, technology, engineering, art and mathematics to culturally rich art exploration and creation. Meanwhile, by applying the learning engagement theory as the model basis, six research hypotheses involving learning engagement (cognitive, emotional and behavioral), continuous improvement attitude and creativity performance were put forward, with a view to understanding the learning effectiveness of and the cognitive and emotional factors affecting course learning in the field of C-STEAM-integrated art design in the context of higher vocational colleges. The results of the current study help to clarify the relationships among students’ learning engagement, their continuous improvement attitude, and their creativity performance during the C-STEAM-integrated Packaging Design course. The experimental results confirmed that learning engagement is positively correlated with creativity performance, learning engagement has a positive effect on continuous improvement attitude, and continuous improvement attitude has a positive effect on creativity performance.

### 6.2 Contribution

Through the design of a C-STEAM course, this study effectively incorporated local culture into teaching practice. The course enables students to continuously explore cultural connotations during creation, internalize the identity of Shangshan Culture, and create highly personalized art works based on local culture by taking multiple presentation forms and combining personal feelings with experience. It also enables students to analyze the historical value of cultural artistry and make good use of cultural characteristics, so that local culture can be protected, utilized, inherited, developed and innovatively transformed. Besides, it leads to the development of characteristic higher vocational teaching, and promotes the high-precision cultivation of art talents, which constitute the practical contribution of this study. Additionally, this study confirmed the role of learning engagement in art design learning outcomes (creativity performance) through the engagement theory-based hypothesis model, and enables researchers in the field to better understand the importance of different learning engagement dimensions to art design learning, which constitutes the theoretical contribution of this study.

### 6.3 Recommendations

This study aimed to develop students’ interdisciplinary design competence and cultural literacy, while examining their cognitive and emotional responses, including learning engagement, continual improvement attitude, and creative performance, to enhance their theoretical knowledge, practical skills, and problem-solving abilities. Through the course, students cultivate integrated design and creative expression across packaging material selection, structural fabrication, and surface decoration (Hong et al., 2020a,b).

Teaching evaluation should remain student-centered, emphasizing active participation and implementing multidimensional assessment from sketching and prototyping to typesetting and final product review, thereby avoiding demotivation caused by one-sided or delayed scoring. Guided by Shangshan Culture, instructors broaden students’ perspectives, encouraging deep exploration and integration of local cultural features into designs, which strengthens their confidence in Chinese national culture. The core objective is to create personalized thematic works with enduring relevance by integrating learning experiences, artistic appreciation, and hands-on practice. Thus, integrating C-STEAM education into higher vocational art design is recommended as an effective approach, extendable to other local cultures such as intangible heritage, linking it with diet, music, dance, martial arts, and customs, to cultivate culturally grounded, high-quality design talents (Zhan et al., 2023).

### 6.4 Limitations and further study

For this study, we chose a practical major course, the Shangshan Culture-based STEAM Packaging Design course for Chinese higher vocational art design students, with a specific practical project as the experimental research topic to deepen the teaching reform. Although the multidimensional relationship among learning engagement, continuous improvement attitude and creativity performance has been discussed, the explanation of why different engagement dimensions produce an effect remains incomplete. In future explorations, qualitative interviews and a multimodal approach can be adopted.

Although several background variables were statistically controlled, the study relied on a single-group design and self-reported measures of learning engagement. Future studies could adopt multi-group or longitudinal designs to further disentangle the role of individual differences in C-STEAM learning outcomes.

Additionally, this study failed to comprehend the possibility of integrating other art design courses into C-STEAM education, or the applicability of selecting undergraduate art students as the experimental subjects. Hence, in future studies, participants of different academic systems and majors can be added to make comparisons, the specific outcomes of course learning can be compared among current students versus alumni, and more experimental courses on art design can be added for comparison.

Finally, based on the Chinese context, the art design education and vocational education need to be researched in line with Chinese policies, including the teaching innovations based on curriculum ideology and politics, technology-enabled art design, cultural education, cultural self-confidence building, and the relationship between art design education and new quality productive forces. Further studies can be carried out in the above areas from different theoretical perspectives.

## Data Availability

The raw data supporting the conclusions of this article will be made available by the authors, without undue reservation.
