# Supplementary material for: The relationships among learning engagement, continuous improvement attitude and creativity performance: a study based on a C-STEAM course
Source: Front Psychol. 2026 Feb 18;17:1738042. doi: 10.3389/fpsyg.2026.1738042 (PMC12956631; doi:10.3389/fpsyg.2026.1738042)
Supplement: Supplementary file 1 [file Table_1.docx]

**Appendix 1**

Teaching objectives

| **Course content** | **Knowledge objectives** | **Skill objectives** | **Cultural inheritance objectives (C)** |
| --- | --- | --- | --- |
| 1. Basics of packaging design  Week 1  (8 class hours) | S: Understand the history of packaging materials such as lacquer, ceramics, glass, metal, paper and plastic.  T: Comprehend the packaging techniques such as printing, die-cutting, creasing, gilding and laminating.  E: Grasp the knowledge of designing external and internal packaging structures.  A: Understand the style and presentation of traditional, modern, nostalgic, simple, cartoon and green packaging designs.  M: Know about the relevant sizes of small, medium and large packages. | S: Can choose appropriate packaging materials by considering the transportation process and display effect of different commodities.  T: Can master a variety of packaging forms.  E: Can comprehensively consider the internal packaging structure from the basic function perspective combined with technical conditions.  A: Can select the appropriate elements and arrangements according to the form and style of packaging.  M: Accurate functional analysis of different sizes of packages. | 1) Appreciate, understand and explore the connotation of Shangshan Culture;  2) Can apply the learned multidisciplinary knowledge of science, technology, engineering, art and mathematics to the exploration of Shangshan Culture-rich concepts and the creation of art works;  3) Strengthen cultural identity, develop national self-confidence, and enhance the ability to promote local culture. |
| 2. Visual communication design of packaging  Weeks 2 to 3  (16 class hours) | S: Understand new composites and traditional natural materials.  T: Grasp the principles of layout design.  E: Become familiar with various paper packaging structures.  A: Comprehend the figurative, abstract and imagery graphics, as well as the textual types on packaging.  M: Know about the most economical, Least wasteful and aesthetic carton material sizes, as well as the size with optimal compressive strength. | S: Grasp the economic and scientific way of selecting packaging materials.  T: Master production techniques such as paper shearing, cutting, folding, inserting and sticking.  E: Become proficient in the design of normal and special form paper structures.  A: Can skillfully apply photography, illustration, cartoon, planar graphics, geometric patterns and logos to packaging decoration.  M: Become proficient in calculating the inner diameter, cutting die size and outer diameter. |  |
| 3. Packaging design strategies and processes  Weeks 4 to 5  (16 class hours) | S: Understand the "3R1D principle" of packaging materials.  T: Comprehend packaging design processes from sketching, computer-aided drafting, draft optimization to printing production.  E: Grasp the matching between outer structure and inner connotation of packaging.  A: Understand packaging decoration to distinguish a brand from others with a unified image.  M: Know about the encoding format and order of barcodes. | S: Can select materials guided by the green concept.  T: Can sketch drafts progressively on the computer, and constantly revise them before mass printing.  E: Innovative design of containers based on the commodity demand.  A: Can skillfully use the graphics, typography and color to highlight the brands.  M: Grasp the standard size and zoom ratio of commodity barcodes. |  |
| 4. Creation of serialized Shangshan product packaging  Weeks 6 to 9  (32 class hours) | S: Can accurately understand the characteristics of serialized packaging materials.  T: Master the design and production process of serialized commodity packaging.  E: Comprehend the design requirements and rules for serialized packaging structures.  A: Understand the positioning design of packaging decoration.  M: Become familiar with the basic dimensions of serialized packaging design. | S: Can choose different packaging materials for different products.  T: Become proficient in computer-aided design skills.  E: Can preliminarily make ergonomic packaging designs.  A: Grasp the information behind different commodities for extracting and summarizing relevant elements.  M: Can determine the specification, capacity, shape and size corresponding to different product forms. |  |

**Appendix 2**

Course unit 3

| Course unit 3: Packaging design strategies and processes | | |
| --- | --- | --- |
| Analysis of Shangshan Cultural elements (C) | A | Based on the shapes of various pottery and stoneware from Shangshan Site, explore the morphological bionic design of paper packaging to enrich the formal languages of shape, structure and function design. |
|  | B | Geometric patterns on pottery and stoneware can be integrated into the paper packaging to enhance aesthetics. |
|  | C | Analyze the pottery and stoneware production methods, and diversify the packaging manufacturing means. |
| Knowledge of material characteristics and selection (S) | A | Reduction of materials focuses on reducing volume in design and saving resources in production to cut energy consumption. |
|  | B | Prioritize the use of reusable materials when designing packaging for beer, soy sauce, beverages, vinegar, etc. |
|  | C | Recycled materials are divided into physical and chemical types, such as extending the packaging service life with plastics and other polymer materials. |
|  | D | Degradable materials emphasize the whole process from raw material selection to waste disposal, the enhancement of environmental protection concept and the strict control of toxic substances. |
| Knowledge of production techniques and tool application (T) | A | Drafts reflect the original intention of designers and record the process of design idea development. |
|  | B | Design drafts are generally produced in the computer at actual size. In addition to the expanded view, the simulation drawing of finished products is also required. |
|  | C | Before formal printing, small-batch trial printing should be carried out for comparison purposes with the design drafts, and to serve as the basis and reference for printing process adjustment. |
|  | D | Designers should personally supervise the printing process, so that they can guide the color fine-tuning of prints to allow mass production. |
| Knowledge of structure and modeling (E) | A | Harmonic contrast between lines and the optimization combination are the main elements shaping the contour of packaging design. |
|  | B | In terms of volume and weight, a basic block is first taken as the prototype, and then combinations are added and subtracted for coordination and unity purposes, thereby forming different structures. |
|  | C | Overall packaging structure is made partially permeable, in order to pursue the modeling aesthetics and meet the actual needs. |
| Knowledge of artistic aesthetics (A) | A | Visual images of the same brand names and trademarks are unified to eliminate the information reciprocity. |
|  | B | The unity of graphic style and the consistency of presentation techniques can effectively highlight the serialized packaging. |
|  | C | By unifying tone or using special brand color as the theme color of serialized packaging, the product type and brand can be identified based on color. |
| Knowledge of packaging dimension (M) | A | Packaging barcode size is 37.29 mm*26.26 mm, and the scaling factor is strictly controlled between 0.8–2.0. |
|  | B | The minimum size of packaging QR codes is controlled at about 19 mm*, and the most appropriate resolution is 150–300 dpi. |

**Appendix 3**

**Chinese Higher Vocational College Students Creative Performance Questionnaire**

Dear students：

Thank you for filling in this questionnaire. At present, research is being carried out on the "The Effects of Art Design Courses in Higher Vocational Colleges Based on C-STEAM". We hope your valuable comments will help us to understand more about this study. Your feedback and basic information is strictly confidential, and there are no right or wrong answers to the questions, so please answer the following questions truthfully.

**Basic information**

1. Name

2. Gender: Man□ Woman□

3. Date of birth

4. Whether to major in art design in high school Yes □ No □

5. The beginning of contact with art design time

kindergarten □ Primary school □ Junior high school □ Senior high school □ College□

6. Have you ever taken a STEAM course? Yes □ No □

7. Have you ever learned Shangshan culture? Yes □ No □

**Sensory feedback**

There are no right or wrong answers to these questions. Don't spend too much time thinking about your answers. Please don't leave anything out. This questionnaire prepared by Hong et al. (2019) and Ye et al. (2020).

| **Question** | | **very**  **disagree** | **disagree** | **maybe** | **agree** | **very**  **agree** |
| --- | --- | --- | --- | --- | --- | --- |
| **Cognitive Engagement** | |  |  |  |  |  |
| 1. | I always have a plan before I design them. | □ | □ | □ | □ | □ |
| 2. | I will remind myself to check again for some things easy to be wrong. | □ | □ | □ | □ | □ |
| 3. | I will be careful not to make mistakes again after making a mistake in my own responsibility. | □ | □ | □ | □ | □ |
| 4. | If there are important details in the design creation, I will record them so as not to forget. | □ | □ | □ | □ | □ |
| 5. | During the design process, I can focus on the key points and never deviate from the topic when discussing with classmates or teachers. | □ | □ | □ | □ | □ |
| 6. | I always dig into the details and reasons of everything. | □ | □ | □ | □ | □ |
| 7. | In the design process, I can always concentrate on the discussion with my classmates or teachers. | □ | □ | □ | □ | □ |
| 8. | When I discuss with my advisor, I can write down key points and headings clearly. | □ | □ | □ | □ | □ |
| **Emotional Engagement** | |  |  |  |  |  |
| 1. | I like to spend time on design work. | □ | □ | □ | □ | □ |
| 2. | I am happy to help my team members. | □ | □ | □ | □ | □ |
| 3. | I often compliment my team members on their performance. | □ | □ | □ | □ | □ |
| 4. | If I make a mistake, I will be brave to admit it to others. | □ | □ | □ | □ | □ |
| 5. | I like to discuss design problems with team members. | □ | □ | □ | □ | □ |
| 6. | If the feature work is not up to the ideal result after completion, I am still willing to revise it again. | □ | □ | □ | □ | □ |
| 7. | In the process of thematic design, I like to take the initiative to explore unfamiliar things. | □ | □ | □ | □ | □ |
| 8. | In the process of thematic design, I will continue to correct and not get angry when I encounter repeated problems in the function of the work. | □ | □ | □ | □ | □ |
| 9. | In the process of designing a topic, when something happens, I prefer to discuss the cause or solve the problem rather than discuss it with my classmates who should be responsible for it. | □ | □ | □ | □ | □ |
| **Behavioral Engagement** | |  |  |  |  |  |
| 1. | When I have to do a project design, I usually arrive at the classroom on time. | □ | □ | □ | □ | □ |
| 2. | I will complete the data collection of the part I am responsible for. | □ | □ | □ | □ | □ |
| 3. | I usually put the design together in the expected time. | □ | □ | □ | □ | □ |
| 4. | I actively participate in thematic design activities. | □ | □ | □ | □ | □ |
| 5. | I have a habit of finishing a project to a certain extent before I take a break. | □ | □ | □ | □ | □ |
| 6. | In order to make the team effective, I will mention less topics that are not related to the work. | □ | □ | □ | □ | □ |
| 7. | I always try to work well with my partners. | □ | □ | □ | □ | □ |
| 8. | I will not sleep or eat to solve problems, if not solved will be upset. | □ | □ | □ | □ | □ |
| 9. | My teammates say I'm an overcommitted workaholic. | □ | □ | □ | □ | □ |
| **Continuous Improvement Attitude** | |  |  |  |  |  |
| 1. | I often think about how to improve the functionality of my designs. | □ | □ | □ | □ | □ |
| 2. | I often think about how to improve the aesthetics of my designs. | □ | □ | □ | □ | □ |
| 3. | I often think about how to improve the durability of my designs. | □ | □ | □ | □ | □ |
| 4. | I often think about how to improve the stability of my design work. | □ | □ | □ | □ | □ |
| 5. | I often think about how to improve the sophistication of my designs. | □ | □ | □ | □ | □ |
| 6. | As soon as I think it could be better, I'll find a way to start improving it immediately. | □ | □ | □ | □ | □ |
| 7. | I don't think any design works are perfect. There are always improvements. | □ | □ | □ | □ | □ |
| 8. | When there is an opportunity to improve my work, I will take it. | □ | □ | □ | □ | □ |
| **Creative Performance** | |  |  |  |  |  |
| 1. | I can draw things I've never seen before (such as aliens). | □ | □ | □ | □ | □ |
| 2. | I can draw a person or an object.。 | □ | □ | □ | □ | □ |
| 3. | I can doodle or draw boxes of patterns. | □ | □ | □ | □ | □ |
| 4. | I can make clip art out of my pictures. | □ | □ | □ | □ | □ |
| 5. | I can take a well-composed photo with an interesting Angle or method. | □ | □ | □ | □ | □ |
| 6. | I can make sculptures or pottery. | □ | □ | □ | □ | □ |
| 7. | I can enjoy a beautiful picture. | □ | □ | □ | □ | □ |
| 8. | I can propose an understanding of a classic work of art. | □ | □ | □ | □ | □ |
| 9. | I enjoy art museums. | □ | □ | □ | □ | □ |

**Thank you for taking the time to fill in the questionnaire! Please confirm again that you have answered each question. Thanks!**
